# The relevance of serum macrophage migratory inhibitory factor and cognitive dysfunction in patients with cerebral small vascular disease

**DOI:** 10.3389/fnagi.2023.1083818

**Published:** 2023-02-07

**Authors:** Jianhua Zhao, Xiaoting Wang, Qiong Li, Chengbiao Lu, Shaomin Li

**Affiliations:** ^1^Henan Joint International Research Laboratory of Neurorestoratology for Senile Dementia, Henan Key Laboratory of Neurorestoratology, Department of Neurology, First Affiliated Hospital of Xinxiang Medical University, Xinxiang, Henan, China; ^2^Sino-UK Joint Laboratory of Brain Function and Injury of Henan Province, Department of Physiology and Neurobiology, Xinxiang Medical University, Xinxiang, China; ^3^Ann Romney Center for Neurologic Diseases, Brigham and Women’s Hospital, Harvard Medical School, Boston, MA, United States

**Keywords:** cerebral small vascular disease, cognitive dysfunction, macrophage migration inhibitory factor, pathogenesis, research progress

## Abstract

Cerebral small vascular disease (CSVD) is a common type of cerebrovascular disease, and an important cause of vascular cognitive impairment (VCI) and stroke. The disease burden is expected to increase further as a result of population aging, an ongoing high prevalence of risk factors (e.g., hypertension), and inadequate management. Due to the poor understanding of pathophysiology in CSVD, there is no effective preventive or therapeutic approach for CSVD. Macrophage migration inhibitory factor (MIF) is a multifunctional cytokine that is related to the occurrence and development of vascular dysfunction diseases. Therefore, MIF may contribute to the pathogenesis of CSVD and VCI. Here, reviewed MIF participation in chronic cerebral ischemia-hypoperfusion and neurodegeneration pathology, including new evidence for CSVD, and its potential role in protection against VCI.

## 1. Introduction

Cerebral small vascular disease (CSVD) is one of the common, chronic, and progressive cerebrovascular disease, accounting for about 25% of ischemic stroke, and it is also an important cause of dementia (Wardlaw et al., 2019). CSVD is caused by various pathological changes of intracranial arterioles, venules, and capillaries, with clinical manifestations of ischemic stroke, dementia, gait disturbance, urinary incontinence, and depression. CSVD primarily affects the small perforating arteries, approximately 100–400 μm in diameter, which supply the white matter and deep structures of the brain, with concentric smooth muscle thickening, as well as pericyte degeneration, basal membrane thickening, endothelial, and astrocyte endfeet swelling in capillaries (Østergaard et al., 2016), causing arteriolosclerosis, and the slowly progressive worsening of microcirculatory structure and function, resulting in white matter hyperintensity (WMH). CSVD is a highly heterogeneous disease that affects nearly all organs (Chojdak-Łukasiewicz et al., 2021), and is greatly influenced by genetic and vascular risk factors. Nearly half of all vascular cognitive impairment (VCI) is potentially caused by CSVD (Skrobot et al., 2018). Therefore, further understanding the relationship between CSVD and VCI, and finding new sensitive and accurate biomarkers will provide a critical theoretical basis for exploring the pathogenesis of CSVD, and may provide novel diagnostic and therapeutic approaches for CSVD.

Macrophage migration inhibitory factor (MIF) is a multifunctional cytokine produced by various cells, such as vascular endothelial cells, smooth muscle cells, and macrophages, plays a key regulatory role in inflammation and immune response, and has a close relationship with asthma, sepsis, cancer, etc. (Sumaiya et al., 2022). Previous studies have shown that MIF is closely involved with stroke and Alzheimer’s disease (AD) (Popp et al., 2009; Wang et al., 2009; Zis et al., 2015), but its relationship with CSVD and VCI is unclear. Therefore, this review mainly focused on the research progress of MIF and its relevance in CSVD and VCI.

## 2. Overview of CSVD and VCI

CSVD is one of the common cerebrovascular diseases, and its prevalence is higher than stroke in the elderly (Cannistraro et al., 2019). The prevalence and incidence of CSVD increase with age. The white matter hyperintensity, the typical imaging feature of CSVD, affects about 5% of people aged 50 years and almost 100% of people older than 90 years. Similarly, cerebral microbleed (CMB) increased from 6.5% in patients aged 45–50 years to about 36% in patients aged 80–89 years (Poels et al., 2010; Moran et al., 2012; Chojdak-Łukasiewicz et al., 2021). The early clinical manifestations of CSVD are insidious, diverse, and changeable, so they are difficult to identify. However, many irreversible effects occur in the advanced stages, such as neurological defects, vascular dementia, urinary and defecation disorders, etc., which can bring great burden to the patients, families, and society. Acute CSVD can rapidly progress to lacunar stroke or intracerebral hemorrhage, while chronic CSVD is mainly associated with progressive cognitive decline, abnormal gait, emotional and sleep disorders, and bowel and bladder disorders (Moran et al., 2012). The main imaging manifestations are recent small subcortical infarct (RSSI), vascular origin lacunae, WMH, perivascular space (PVS), CMB, and brain atrophy (Wardlaw et al., 2013b, 2019), which can exist alone or in combination and have different effects on cognitive function (Meng et al., 2019).

VCI refers to cognitive impairment caused by various cerebrovascular diseases, principally infarction in cortical and subcortical and extensive white matter damage due to CSVD (Banerjee et al., 2016). CSVD is one of the common causes of VCI, which is usually associated with the progressive decline of cognitive function, and can lead to the emergence of new cognitive impairment (CI) (Azeem et al., 2020). Irrespective of stroke, CSVD will lead to CI, and is often insidious and atypical in early stage (van Uden et al., 2016; Wolters and Ikram, 2019). CI caused by CSVD accounts for 36–67% of all vascular dementias (VaD) (Peng, 2019) and 15–30% of all dementias, second only to AD (Boyle et al., 2018; Wardlaw et al., 2019; Wolters and Ikram, 2019). Recent research shows that CI could account for 65.0% of all CSVD cases, of which 40.0% have mild cognitive impairment (MCI). So it is important to identify CI as early as possible. The number of people suffering from dementia is expected to reach 100 million by 2050 (De Silva and Faraci, 2020). With increasingly aging population, CSVD-CI will inevitably bring huge challenges to public health and economic development.

## 3. Overview of macrophage migration inhibitory factor

In 1966, the migration inhibitory activity of MIF was first reported by Bloom and Bennett (1966) in a delayed-type hypersensitivity study. In 1993, MIF was identified to be a secreted pro-inflammatory protein, and the physiological and pathological characteristics of MIF and its receptors were subsequently elucidated (Bernhagen et al., 1993). MIF consists of 114 amino acids and is an evolutionarily highly conserved low molecular homotrimeric protein (about 12.5 kDa). The MIF gene is located on chromosome 22 (22q11.23) of the human genome, containing three exons and two introns (Sun et al., 1996). The MIF gene has polymorphisms of transcription factors and the promoter region, and these can significantly affect the transcription of MIF gene, which determines its ability to modulate susceptibility and severity of infectious and autoimmune diseases (Baugh et al., 2002; Radstake et al., 2005).

MIF is widely expressed in various types of cells and tissues, including immune and nervous system cells, pituitary cells, epithelial cells, endothelial cells, smooth muscle cells, etc., and it is highly expressed in the nervous system, mainly in the cortex layer (Michell-Robinson et al., 2015). Studies found that MIF is abundantly expressed in nerve cells such as astrocytes, microglia, oligodendrocytes, neurons, and Schwann cells (Su et al., 2017). The concentration of MIF in cerebrospinal fluid is similar to serum (Ogata et al., 1998). The secretion of MIF is mainly regulated by the hypothalamic–pituitary system, and glucocorticoids are also involved in regulation (Fan et al., 2014). Different from the classical synthesis and secretion of other factors, MIF is abundantly stored as a precursor in the cytoplasm. After stimulation by endotoxin, ischemia, hypoxia, etc., MIF is directly, massively, and rapidly released to exert potent biological functions, and has an inflammation magnification effect (Dayawansa et al., 2014).

MIF is involved in various biological functions including leukocyte recruitment, inflammation, immune response, cell proliferation, tumorigenesis, and regulation of glucocorticoids. Its unique pathological roles can be involved in different diseases (i.e., sepsis, rheumatoid arthritis, diabetes, malignant tumor, acute respiratory distress syndrome, hepatitis, and systemic lupus erythematosus) (Sumaiya et al., 2022). MIF has also been reported to play numerous roles in neurological disorders. Clinical studies have confirmed a close relationship between MIF and atherosclerosis (AS), during which MIF can accelerate AS through immune reaction, inflammation, and oxidative stress, and promote neuronal death after stroke, and thus aggravate the development of stroke (Grieb et al., 2010; Li et al., 2017). MIF is associated with biomarkers of AD pathology and predicts cognitive decline in MCI and mild dementia (Oikonomidi et al., 2017). Mittelbronn et al. (2011) found that MIF expression was increased in astrocytes compared with the normal control group, and the expression was significantly positively correlated with tumor tissue grade.

## 4. Macrophage migration inhibitory factor and CSVD

CSVD is believed to be a dynamic disorder of the brain. The abnormal function of neurovascular unit (NVU) plays an important role in CSVD (Iadecola, 2017). The pathogenesis of CSVD includes chronic ischemia and hypoperfusion, endothelial dysfunction (ED), blood–brain barrier (BBB) damage, interstitial fluid reflux disorder, inflammatory and genetic factors, etc., as well as some shared mechanistic interactions. CSVD is a small ischemic or bleeding lesion caused by pathological small vessels or brain degeneration (Wardlaw et al., 2013b), and is an important cause of ischemic stroke. In order to identify detection and treatment targets, studies have detected the lineage changes of various cytokines/chemokines in the plasma of ischemic stroke patients, and confirmed that MIF is significantly elevated (Liu et al., 2018). Given that CSVD is also a chronic ischemic cerebrovascular disease, pro-inflammatory cytokines and chemokines are elevated during ischemia caused by CSVD, which promotes post-ischemic inflammation and leads to neuronal damage. CSVD has similar etiology and pathological mechanisms as hypoxia, inflammation, and immunoreaction. It is speculated that MIF may act on CSVD through different pathogenesis and become a reliable target for the detection and treatment of CSVD.

### 4.1. MIF and chronic cerebral ischemia-hypoperfusion

Chronic cerebral ischemia-hypoperfusion plays a key role in the pathogenesis of CSVD, especially in arteriosclerosis. Similar to risk factors for macrovascular stroke, common factors in CSVD include hypertension, diabetes, hyperlipidemia, and hyperhomocysteinemia. These factors can stimulate systemic inflammation and promote arteriolosclerosis (Ighodaro et al., 2017), and are also associated with WMH, PVS, and CMB (Verhaaren et al., 2013). Hypertension and age are the most important independent factors (Gyanwali et al., 2019), which can cause microvascular damage, increase in the medial-lumen diameter ratio, and decrease in cerebral blood flow (CBF), resulting in hypoxia, BBB leakage, inflammation, edema, a cascade of NVU dysfunction, and ischemia caused by oligodendrocyte impairment (Wardlaw et al., 2013a; Mestre et al., 2017; Evans et al., 2021). Studies have shown CBF reduction of white matter in CSVD. Local low CBF is significantly negatively correlated with BBB permeability (Bailey et al., 2012). Patients with lower CBF usually had more WMH (O'Sullivan et al., 2002; Shi et al., 2016), and WMH may be a predictor of vascular cognitive dysfunction (Meng et al., 2019). Moreover, CBF reduction also occurred in normal white matter surrounding the WMH, which is possibly related to future WMH expansion (Promjunyakul et al., 2015). Both WMH and white matter segmented as normal-appearing by structural MRI exhibited BBB damage and hypoperfusion, which increased near the WMH and were correlated.

Arteriosclerosis may be a common pathogenesis of CSVD (Nam et al., 2020). Hypertension is associated with or preceded by arterial stiffening (Webb and Werring, 2022). Stiffened arterioles lose autoregulation in the brain, and chronic exposure to high-fluctuating pulse energy in arterioles can cause damage to the vessel wall, leading to ED and fatty hyaluronan deposition, which disrupts blood–brain barrier integrity, promotes neuroinflammation, and may contribute to amyloid deposition and Alzheimer pathology (Santisteban et al., 2023). In addition, the loss of resistance can maintain the diastolic blood pressure lower than normal, resulting in chronic hypoxia of the brain. Studies have shown that even in the absence of intracranial vascular stenosis, arteriosclerosis detected by MRI may be an important risk factor for WMH (Kim et al., 2014). Arteriosclerosis has been shown to be independently associated with WMH, overall cognitive function, and an increased risk of AD and dementia (Saji et al., 2011; Arvanitakis et al., 2016; Ighodaro et al., 2017).

Some cross-sectional investigations have underscored that subclinical atherosclerosis and arteriosclerosis often coexist (Vishnu et al., 2015; Kim and Kim, 2019), and both arterial stiffening and plaque formation depend partly on the same systemic pathophysiological process causing the accumulation of extracellular matrix in the arterial walls (Lee and Oh, 2010). Dysfunctional endothelium and increased vascular stiffness are the main features of preclinical atherosclerosis, and stiffened vessels supply the environment for vascular disease progression, and are regarded as an independent predictor of cardiovascular disease events (Laurent et al., 2006). Clinical evidence indicates an association of MIF plasma levels with diminished endothelial function and increased vascular stiffness in patients with established cardiovascular risk (Rammos et al., 2013). MIF is involvement in the preclinical atherosclerosis process based on low-grade inflammation (Schober et al., 2008), has pro-inflammatory and pro-atherogenesis functions, and has become the main regulator of atherosclerosis (Asare et al., 2013).

Previous studies have found that MIF promoter activity is significantly up-regulated under hypoxia (Zis et al., 2015), and MIF is elevated in ischemic stroke in rodent models and patients (Wang et al., 2009; Zis et al., 2015; Liu et al., 2018), is associated with stroke clinical severity (Yang et al., 2017), and could predict severity and prognosis in patients with ischemic stroke (Li et al., 2017; Wang et al., 2019). Lin et al. (2000) first demonstrated that MIF is significantly up-regulated during AS, which promotes macrophage aggregation, infiltration, proliferation, and activation, enhances macrophage phagocytosis, and mediates inflammatory damage to brain tissue after hypoxia (Grieb et al., 2010). MIF is a key mediator of AS, promoting leukocyte recruitment and inflammation, and is involved in the entire development (Burger-Kentischer et al., 2002). Furthermore, MIF expression by macrophages may initiate and amplify AS process (Lin et al., 2000). In the MIF-knockout mice, the inflammation in AS was reduced, preventing further thickening of the arterial intima (Pan et al., 2004). Studies have shown that MIF has a detrimental effect in permanent cerebral ischemia under hypertensive conditions, in which MIF can specifically aggravate the loss of vascular integrity after stroke. The MIF antagonist ISO-1 plays a protective role in ischemic stroke (Liu et al., 2018), after the neutralizing function is effective and the expression of MIF in inflammatory cytokines was suppressed (Lan et al., 1997). However, some studies have shown that MIF exerts neuronal protection (Kim et al., 2022), and down-regulation of MIF in hypoxic conditions accelerates neuronal damage during stroke. Furthermore, MIF reduced the activation of caspase-3 (the critical terminal cleavage enzyme in apoptosis) and protected neurons from oxidative stress and ischemia/reperfusion-induced apoptosis *in vitro* (Zhang et al., 2014). MIF knockout mice showed activated caspase-3, neuronal loss, and infarct development during stroke. The broad spectrum of MIF’s actions and the complexity of MIF expression in the brain post-stroke, challenge the identification of the mechanism or mode of action of MIF in cerebral ischemia.

### 4.2. MIF, endothelial dysfunction and blood–brain barrier disruption

In the central nervous system (CNS), endothelial cells (ECs) are the main structures that constitute the NVU and BBB, and play an important role in maintaining vascular morphology and biological function (De Silva and Faraci, 2020). The BBB only allows water and small-molecule lipid-soluble substances to diffuse freely due to a concentration gradient, and it is critical in maintaining the homeostasis of the internal environment of the CNS. Studies have shown that BBB dysfunction is an important pathogenesis of CSVD (Wardlaw, 2010), and the increased BBB permeability is associated with higher white matter overload and cognitive decline (Walsh et al., 2021), and is an early biomarker of cognitive decline (Nation et al., 2019). Recent studies have shown that ED may be the initiating factor of CSVD, prior to other pathological mechanisms (Rajani et al., 2018), and can cause vascular wall lipid hyaline degeneration, toxic damage to brain parenchyma nerve cells, etc. (Hainsworth and Fisher, 2017; Quick et al., 2021) leading to lacunar infarction (Pretnar-Oblak et al., 2006). Studies have shown that the integrity of ECs declines with age, leading to a decline in BBB function (Montagne et al., 2015), which may be a potential reason for the high incidence of CSVD in the elderly (Farrall and Wardlaw, 2009). Studies have shown that pericyte dysfunction could act on BBB, angiogenesis, and CBF (Uemura et al., 2020). In pathological conditions of ischemic cerebrovascular disease (Hall et al., 2014) and AD (Nortley et al., 2019), the contraction of pericytes leads to capillary constriction and narrowing, resulting in a decrease in CBF, and amyloid β beta (Aβ) clearance disorders, which are thought to be key factors for aggravating dementia diseases such as VCI and AD (Montagne et al., 2018). Studies have shown that MIF is related to BBB damage. ECs express MIF receptors and MIF can induce ECs autophagy, leading to ED and increased vascular permeability (Chen et al., 2015). It is known that MIF promote the production of pro-inflammatory cytokines including tumor necrosis factor-α (TNF-α), interleukin-1(IL-1), and interleukin-6(IL-6), which has been reported to increase BBB permeability (Sandoval and Witt, 2008). In an *in vitro* study on primary cortical cells and an *in vivo* study in an animal model of middle cerebral artery occlusion (pMCAo), MIF did not produce direct toxicity in primary culture, but disrupted tight junctions of ECs (Liu et al., 2018). Administering MIF after pMCAo can damage the tight junction of the BBB, increase the infarct size, and severely impair neurological function, leading to a deleterious effect on stroke. In addition, ISO-1 has a strong neuroprotective effect. These findings suggested that MIF may be a target for the treatment of stroke. Studies have found that perivascular macrophages could produce a large amount of superoxidase and reactive oxygen species by increasing brain barrier permeability, thereby causing neurovascular damage and cognitive dysfunction associated with hypertension (Faraco et al., 2016; Santisteban et al., 2020). While under hypoxic conditions caused by arteriosclerosis, a large amount of MIF stored in macrophages is activated and released, triggering a series of inflammatory and immune responses, which may be closely related to cognitive damage caused by CSVD.

### 4.3. MIF and inflammation

Inflammation is involved in the overall process of CSVD. Many mechanisms, including ED, BBB damage, arteriosclerosis, and white matter lesions, are all associated with inflammation (Evans et al., 2021). A potential cause of arteriosclerosis involved in age and hypertension is considered the chronic low-grade inflammation of the vessel wall. The upregulation of the inflammatory response is the consequence of a remodeling of the innate and acquired immune system with a chronic inflammatory cytokine production (Baylis et al., 2013). Chronic inflammation directly influences premature atherosclerosis and arterial stiffness (Roman et al., 2005), probably plays an important role in triggering fibrosis in cardiovascular disease and hypertension. ED can destroy BBB, increase its permeability, and allow peripheral lymphocytes to enter the brain to produce an immune response to CNS antigens, promote the infiltration of inflammatory cells into the lesion and surroundings, and even participate in the immune response, thus aggravating tissue damage. Meanwhile, fibrinogen enters the CNS and is converted into fibrin, which in turn activates microglia and macrophages, leading to oligodendrocyte and neuronal damage, resulting in chronic inflammatory microenvironment in the extracellular matrix and NVU, and exacerbating CSVD. In addition, fibrinogen can inhibit the expression of peroxisome proliferator-activated receptor in smooth muscle cells, resulting in increased expression of C-reactive protein and MMP-9, and accelerated progression of AS (Wang et al., 2015), while high MMP-9 was found to be an important risk factor for non-dementia VCI. Arteriolosclerosis causes chronic ischemia-hypoperfusion and massive inflammatory reactions, and the resulting lacunar infarction may also activate inflammation due to necrosis of brain tissue (Lambertsen et al., 2019; Al Mamun et al., 2020), further leading to acute or chronic brain damage.

**Figure 1:**
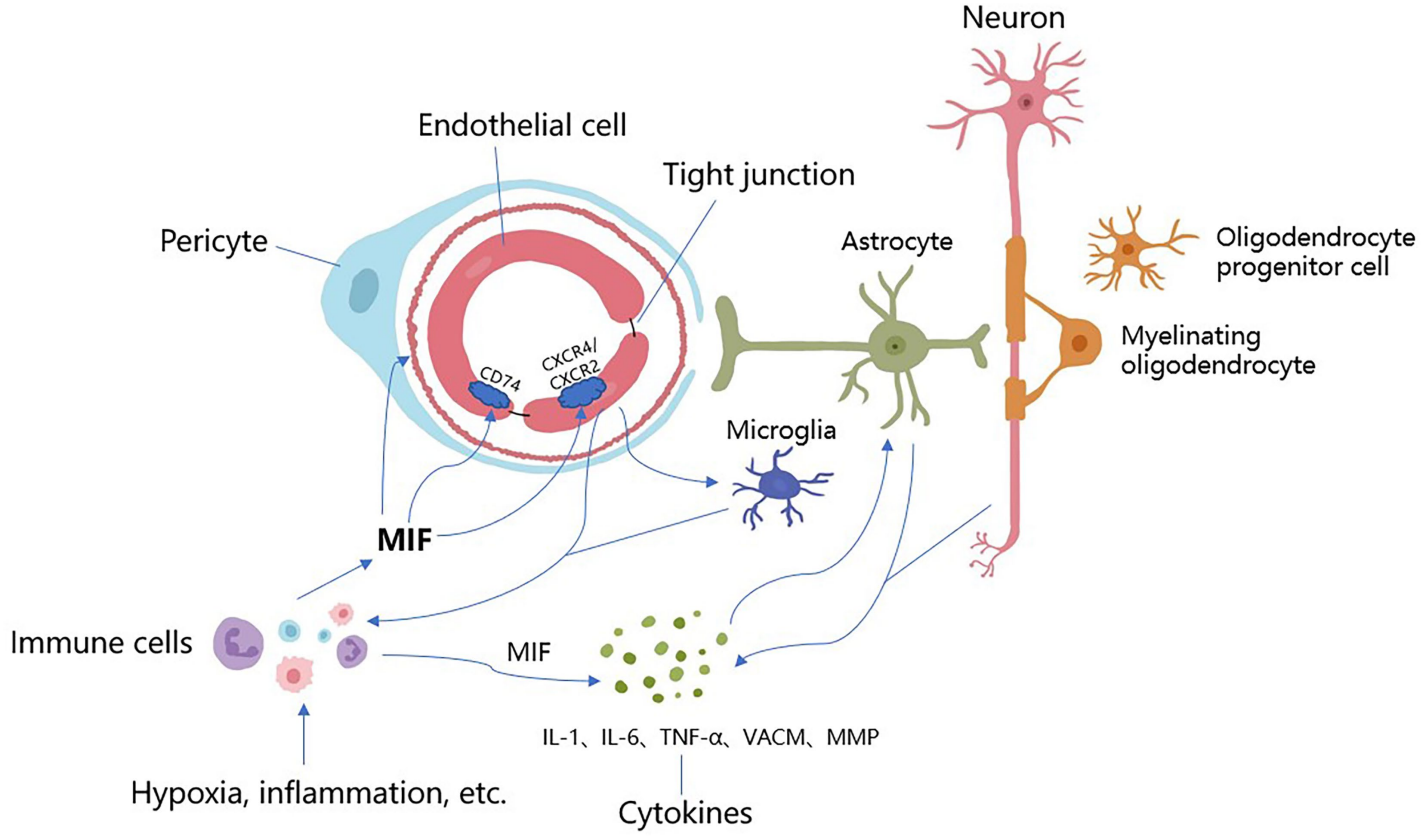
The underlying mechanisms of MIF acting on CSVD. Stimulated by hypoxia, inflammation and other factors, MIF is expressed in large quantities from immune cells (i.e., mononuclear, macrophages), which promotes arteriolosclerosis, narrows the lumen and reduces cerebral blood flow, resulting in further hypoxia and ischemia of the brain parenchyma. After binding to receptors, MIF causes endothelial autophagy, promotes endothelial cell dysfunction and BBB destruction, increases vascular permeability, allows harmful components and immune cells to enter the brain parenchyma, and causes neuroinflammation and direct neurotoxic effects. Meanwhile, MIF activates immune cells and microglia, promotes the expression of MIF, activates a series of cytokines (IL-1, IL-6, TNF-α, VCAM, MMP, etc.), accelerates inflammatory response, induces vascular damage, and destroys the integrity of the blood–brain barrier and white matter. These inflammatory factors directly stimulate astrocytes and neurons to produce more cytokines, promoting further BBB damage and inflammation. There are overlapping reactions between various mechanisms.

MIF has pro-inflammatory effects in local and systemic inflammation (Calandra and Roger, 2003), and may severely affect the inflammatory response under pathological conditions (Denkinger et al., 2004; Liu et al., 2018). It is an inflammatory mediating factor that can be secreted by various cells such as monocytes and macrophages, and can mediate the recruitment of monocytes, neutrophils, and T lymphocytes through non-homologous interactions with chemokine receptor-2 and chemokine receptor-4 (Bernhagen et al., 2007; Schmitz et al., 2018), thereby promoting the expression of cytokines such as TNF-α, IL-1, interleukin-8(IL-8), intercellular adhesion molecule-1(ICAM-1), vascular cell adhesion molecule-1(VCAM-1), etc., which promote leukocyte–endothelial cell interactions (Cheng et al., 2010), and exert an inflammatory effect during AS. These cytokines can also lead to the development of CSVD by increasing the permeability of the BBB (Calandra and Roger, 2003; Sandoval and Witt, 2008). Previous studies showed that the increase of MIF was stronger than other cytokines, suggesting that it plays a regulatory role in the inflammation in stroke (Liu et al., 2018). MIF levels have been found to be highly expressed in human active multiple sclerosis lesions, and MIF plays an upstream mediating role in many neuroinflammations involving autoimmune (Cox et al., 2013). Some studies have also shown that MIF-treated rats have increased gliosis in the ipsilateral cerebral hemisphere peri-infarct area, where astrocytes produce varied pro-inflammatory cytokines, which can aggravate ischemic injury (Barreto et al., 2011; Neuhaus et al., 2014). While ISO-1 could suppress inflammation (Al-Abed and Van Patten, 2011), in ISO-1-treated rats, there were fewer cells stained for astrocyte activation markers in the brain after stroke (Liu et al., 2018) (See Figure 1).

## 5. MIF and cognitive dysfunction

MIF is a pro-inflammatory lymphokine with broad immune and inflammatory biological activities that can cause CI through multiple mechanisms. CSVD is a small ischemic or bleeding lesion caused by pathological small vessels or brain degeneration. MIF can promote CI through vascular risk factors and neurodegenerative lesions. Previous studies showed that MIF can bind β-amyloid with potentially important pathophysiological implications for the accumulation of Aβ in AD, and MIF co-localizes with microglia surrounding amyloid plaques in AD brains (Oyama et al., 2000). Studies have shown that serum MIF levels are higher in MCI and AD than in the normal control groups (Lee et al., 2008).

MIF plays a central role in the regulation of microglial activation and inflammatory accumulation, caused by local inflammation in AD brains, and is associated with amyloid pathology, tau hyperphosphorylation, and neuronal damage in early stages of AD, and also exerts pro-inflammatory effects on AD (Oikonomidi et al., 2017). MIF levels are significantly elevated in the cerebrospinal fluid in MCI and AD, and higher MIF levels are associated with accelerated cognitive decline in MCI and mild dementia (Oikonomidi et al., 2017; Nasiri et al., 2020). The neuroinflammation mediated by MIF may persist in all clinical stages of AD (Popp et al., 2009). In experimental models of AD, attenuation of MIF inhibits astrocyte activation and tau hyperphosphorylation (Li et al., 2015). In a cell culture study conducted by Bacher, blocking MIF using ISO-1 significantly reduced Aβ-mediated neurotoxicity, suggesting direct effects of MIF on microglia (Bacher et al., 2010).

However, AD mouse model experiments conducted by Nasiri et al. (2020) showed that MIF improved cognitive function by down-regulating the production of pro-inflammatory cytokines, demonstrating that MIF deletion has a protective effect on spatial learning defects. Studies have also shown that pro-inflammatory stimuli can significantly up-regulate MIF in the hippocampus, which may be related to CI in schizophrenia patients, who have significantly reduced abilities in daily work and memory (Chai et al., 2020). In an *in vitro* model of Parkinson’s disease (PD), overexpression of MIF could protect dopaminergic neurons and reduce neuronal neuroinflammation, while knockout of MIF in an AD mouse model could impair cognition, suggesting that MIF may be involved in the process of PD and even PD-CI (Li et al., 2019; Zhang et al., 2019). Therefore, the effect and mechanism of MIF on cognitive function need to be further studied.

**Figure 2:**
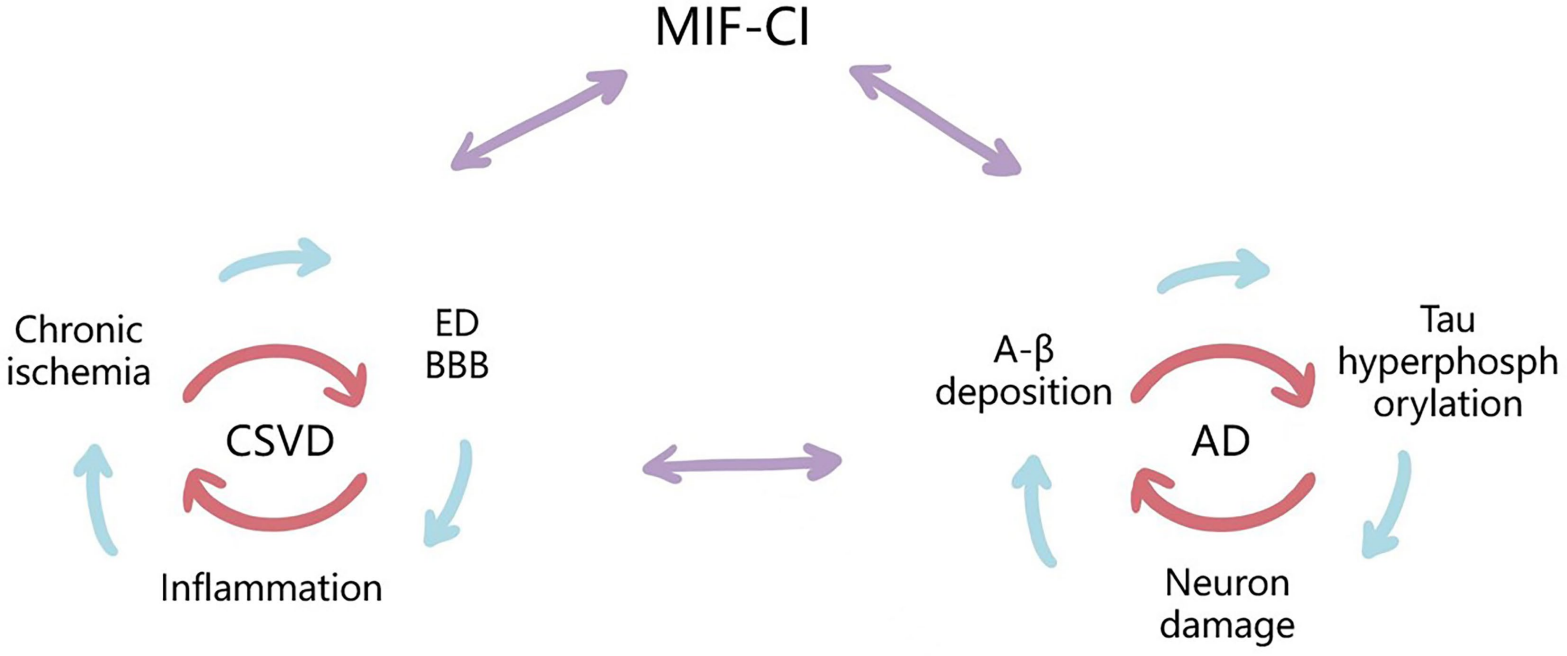
Possible mechanisms for the Upregulating Role of MIF and Synergism Between CSVD-CI and AD-CI. MIF may cause cognitive impairment through both vascular risk factors and neurodegenerative lesions. CSVD is a small ischemic or bleeding lesion caused by pathological small vessels or brain degeneration, including chronic cerebral ischemia and hypoperfusion, ED and BBB destruction, inflammatory response, and other pathogenesis, which are interrelated with the pathologies of AD, and MIF may be involved in the above mechanisms. They all cause neuronal and cognitive impairment by initiating a cascade of inflammatory cytokines.

It is generally accepted that mixed pathologies (coexistence of cerebrovascular diseases and neurodegenerative pathologies) are currently an important factor in the development of AD and other forms of dementia (Kapasi et al., 2017). Many studies indicated a complex relationship between AD and cerebrovascular disease, although the initiating factors of neuronal degeneration are different, they both lead to neuronal damage by initiating the cascade reaction of inflammatory cytokines (Wada-Isoe et al., 2004). Studies have shown that CSVD can promote the occurrence and development of AD by increasing the expression of Tau protein (Laing et al., 2020), and arteriolosclerosis and WMH have been shown to be associated with an increased risk of AD and dementia (Arvanitakis et al., 2016; Bos et al., 2018). MIF may cause CI through different pathological mechanisms, which also provides more ideas for the diagnosis and treatment of VCI based on inflammatory factors (See Figure 2).

## 6. Summary and outlook

With the increase in population aging worldwide, CSVD and CI impose a significant burden on individuals and the society. Since the pathogenesis remains unclear and diagnosis of CSVD-CI remains controversial, the search for sensitive and accurate biomarkers will provide new scientific ideas. Many studies have shown that MIF plays a crucial role in ischemic stroke, AD, and other diseases. Given that CSVD and ischemic stroke, and VCI and AD have similar pathogenesis, MIF may be involved in multiple pathogenesis of CSVD leading to VCI, and MIF can cause CI through AD-related pathological processes. Hence, MIF may be a promising biomarker for predicting and treating CSVD and VCI. However, the underlying pathogenesis of CSVD and VCI remains unclear and needs further study.

## Author contributions

JZ and XW designed and drafted the outline of the review, coordinated the writing process, evaluated the quality of the included studies, wrote the manuscript. QL did the literature search. CL and SL provided insight, context, and balanced interpretation of evidence through discussion. All authors contributed to the article and approved the submitted version.

## Funding

National Natural Science Foundation of China (81771517), Natural Science Foundation of Henan Province (182300410389), and Joint construction project of Henan Medical Science and technology research plan (LHGJ20190437).

## Conflict of interest

The authors declare that the research was conducted in the absence of any commercial or financial relationships that could be construed as a potential conflict of interest.

## Publisher’s note

All claims expressed in this article are solely those of the authors and do not necessarily represent those of their affiliated organizations, or those of the publisher, the editors and the reviewers. Any product that may be evaluated in this article, or claim that may be made by its manufacturer, is not guaranteed or endorsed by the publisher.
